#  Serum albumin levels monitoring in ICU in early days and mortality risk association in patients with moderate to severe COVID-19 pneumonia

**DOI:** 10.12669/pjms.38.3.4154

**Published:** 2022

**Authors:** Mirza Ayub Baig, M. Mohsin Raza, Mougheesa Baig, M. Usman Baig

**Affiliations:** 1 Dr. Mirza Ayub Baig (PI) Assistant Professor Pulmonology, Consultant Pulmonologist, Lahore Care Hospital, Al-Shafi Hospital, Lahore, Pakistan. Fatima Jinnah Medical University, Sir Ganga Ram Hospital, Lahore, Pakistan; 2 Dr. M. Mohsin Raza, Consultant physician Sir Ganga Ram Hospital, Lahore, Pakistan; 3 Dr. Mougheesa Baig Medical Officer Lahore Care Hospital, Lahore, Pakistan; 4 Mr. M. Usman Baig, MBBS Student, Ameer-ud-din Medical College, Lahore, Pakistan

**Keywords:** Intensive Care Unit (ICU), C-reactive protein (CRP), Serum Albumin level, COVID-19, Reverse transcriptase polymerase chain reaction (RT-PCR)

## Abstract

**Objective:**

To establish correlation between serum albumin during early days of ICU admission and risk of death in COVID-19 pneumonia.

**Methods:**

In this retrospective study, we included 76 patients hospitalized in ICU, who stayed for at least four days with COVID-19 pneumonia, from May 1, 2020 to June 30, 2020 in Lahore Health Care Hospital and Al-Shafi Hospital. Patients were labelled as COVID-19 pneumonia on radiological basis as bilateral ‘ground-glass opacity’ in lower zones and RT-PCR positive result in nasopharyngeal swab. All patients were oxygen dependent, either on high flow oxygen via non rebreathing mask or invasive positive pressure ventilation support. Serum albumin levels were measured daily from first day to fourth day of ICU admission. The data was analyzed using SPSS version 26 and Microsoft excel 2016.

**Results:**

Out of 76 patients of COVID-19 pneumonia admitted in ICU who stayed for more than four days, 38 patients expired. The mean age of all the patients was 58.9±12.56 years, 38(50%) of the patients were ≥60 years and 49 (62%) of them were male. On day four of ICU admission, mean serum albumin of discharged patients was 3.83±0.22 g/dl while mean serum albumin level of expired patients was 2.96±0.46 g/dl. Strong negative correlation (r = -767) was found between decrease in serum albumin level and increase number of deaths from COVID-19 pneumonia. Weak correlation was observed between increase in serum CRP and increase number of deaths in the same patients.

**Conclusion:**

Daily monitoring of serum albumin level of COVID-19 pneumonia patients can be used as a biological marker for monitoring of cytokine storm and risk of death in COVID-19 pneumonia.

## INTRODUCTION

COVID-19 virus is highly contagious virus due to its ability to transmit itself before development of symptoms.^1^ It has been declared as pandemic by WHO because it is affecting a huge population of the world.^2^ Due to emergence of new strains, number of active cases declined but are on the rise again leading to increase number of deaths in Pakistan and other regions of the World.^2^ This virus affects mainly respiratory system, and Pneumonia is the most common sequelae of COVID-19 disease.^3^ As currently no effective antiviral drug is available so viral pneumonia proceeds to ARDS and respiratory failure.^4^ Ultimately these patients are treated by high flow nasal cannula and invasive positive pressure ventilation. Despite all ventilatory strategy, COVID-19 pneumonia is associated with high mortality.^5^ Various comorbid conditions are being proposed including old age, diabetes mellitus etc.^6^

Similarly, different biomarkers are being proposed including high serum CRP level and LDH due to cytokine storm associated with COVID pneumonia.^7^We noticed in our patients, who had normal or near normal serum albumin level on arrival in ICU, developed hypoalbuminemia within four days in ICU due to cytokine storm. The objective of the study was to evaluate the sudden decrease in serum albumin level in four days just after shifting in ICU as a consequence of cytokine storm and its association with respiratory failure and death. We concentrated on serum albumin monitoring in early days to avoid effect of malnutrition due to prolonged ICU stay.

## METHODS

In this retrospective case-control study, we included patients hospitalized in ICU with COVID-19 pneumonia from May 1, 2020 to June 30, 2020 in Lahore Care Hospital and Al-Shafi hospital. Both the hospitals are recognized by Punjab Health Care Commission. Each ICU has central Oxygen supply, ICU beds, Ventilators, BIPAP machines, Cardiac monitors and round the clock dedicated staff.

### Ethical clearance

The study was approved by the institutional review board of Lahore Care Hospital via letter No.01-Art/ICU-IRB/LCH dated 15-8-2020 and Al-Shafi Hospital via letter No.01-Art/ICU-IRB/ASH (dated:15-8-2020).

### Inclusion criteria

All patients were oxygen dependent, either on high flow oxygen via non rebreathing mask or invasive positive pressure ventilation support.

### Exclusion criteria:

1. Patents who stayed in ICU for less than four days.
2. Those patients who were on oxygen for more than 24 hours before admission in ICU were excluded from the study.
3. Those patients who had CLD, Nephrotic syndrome and Diabetic Nephropathy were excluded from study.
4. Those patients who had blood TLC >10,000 were also excluded from the study.

### Operational definitions:

### COVID-19 pneumonia

Patients were labelled as COVID-19 pneumonia on radiological basis as bilateral ‘ground-glass opacity’ in lower zones with RT-PCR positive result in nasopharyngeal swab.^8^,^9^

### Moderate COVID-19 pneumonia

Patient who maintained SPO2>90% on 10-15 L/minutes oxygen is described by WHO as severe covid-19 disease.^10^

### Severe COVID-19 pneumonia

Patient required more than 15 L/minutes oxygen and, invasive or noninvasive positive pressure ventilator support is described by WHO as critical covid-19 disease.^10^

### ICU admission criteria:

1. Patients who required >10 L/min oxygen or high flow oxygen.
2. Patients who required noninvasive ventilatory support
3. Patients who required invasive ventilatory support.

Serum albumin levels were measured using chemiluminescence immunoassay technique in laboratory. We reviewed the files of patient admitted in ICU from 1 May to 30 June and selected the patients according to inclusion criteria. Then we noted serum albumin level from day one through four from lab record.

### Statistical analysis

For statistical analysis, independent sample t-test and point-biserial correlation was used. The data was analyzed using SPSS version 26 and Microsoft Excel 2016. P-value between mean serum albumin of discharged and expired patients was calculated using Two sample T-test. Correlations were found using point-biserial correlation.

**Fig.1:**
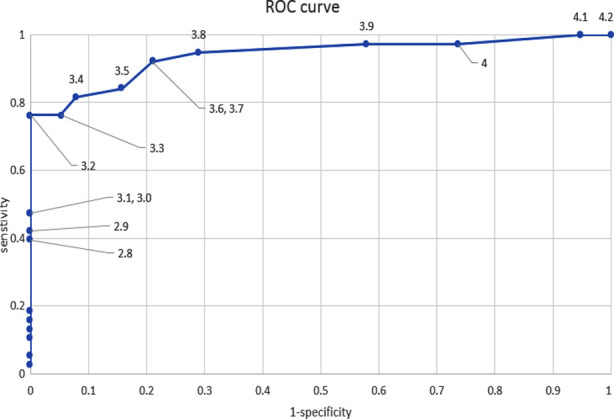
ROC curve to find cut off value of serum albumin level on day 4 of admission in ICU with death taken as sensitivity (y-axis).

## RESULTS

We included 76 patients in this study out of which 38 were survivors. Patient’s demographics and outcomes are summarized in Table-I. The mean age of all the patients was 58.9±12.56 years, 38 (50%) of the patients were ≥60 years of age while 38 (50%) patients were <60 years of age. 49 (62%) patients were male. The P-value for serum albumin levels on day four post admission in ICU between survivors and expired is <0.001 measured using two sample T-test.

**Table 1:** Serum albumin levels of COVID-19 pneumonia patients on day four post admission in ICU. P-value was calculated using two sample T-test on day four of admission in ICU for survivors and expired patients.

Gender	Survivor	Survivor (mean albumin day four “g/dl”)	Expired	Expired (mean albumin day four “g/dl”)	P-value
***Males***					
≤60years	11	3.90±0.16	15	3.08±0.44	<0.001
>60 years	10	3.87±0.17	12	2.98±0.41	<0.001
***Females***					
≤60 years	6	3.8±0.18	5	3.16±0.35	0.002
>60 years	11	3.63±0.24	6	3.08±0.54	0.005

The mean serum albumin level of discharged patients on day one of admission in ICU was 3.68±0.29g/dl (CI 95% [3.58,3.78]) with nine (23%) patients having serum albumin <3.5g/dl. The mean serum albumin level of expired patients on day one of admission in ICU was 3.58±0.31 g/dl (CI 95% [3.48,3.68]) with thirteen (34%) patients having serum albumin level <3.5g/dl. When compared to day four of post ICU admission, only three (7%) patients who were discharged had serum albumin level <3.5g/dl while 31 (81%) of the expired patients had serum albumin level <3.5g/dl. Patients having mean serum albumin <3.5g/dl are summarized in Table-II.

**Table II:** Means and serum albumin levels of patients on Day one to four of ICU admission. P-value measured by comparing serum albumin level of expired and discharged patients using two sample T-test.

Day of ICU admission	Discharged patients mean albumin (g/dL)	Expired patients mean albumin (g/dL)	Serum albumin <3.5g/dl (% of total discharged)/ expired (% of total expired)	P-value
Day 1	3.68±0.29	3.58±0.31	9(23%)/13(34%)	0.07
Day 2	3.62±0.31	3.52±0.32	11(28%)/15(39%)	0.08
Day 3	3.6±0.28	3.43±0.27	8(21%)/20(52%)	<0.001
Day 4	3.8±0.21	3.05±0.42	3(7%)/31(81%)	<0.001

Mean serum albumin level of survivors and expired patients are summarized in Table-II. When the albumin levels of discharged patients were compared to that of expired patients, the p-value came out 0.07, 0.08, <0.001, <0.001 for day one, two, three, four respectively.

Strong negative correlation (r=-0.767) was found between decreased serum albumin on day four and increased risk of death from COVID-19 pneumonia. This correlation was found using point-biserial correlation. An ROC curve was constructed to find cut off value and compare relative risk of death in COVID-19 pneumonia.

Serum C-reactive protein level of expired patients were 134±87.0 and 43±50.4 on day one and day four post ICU admission respectively while discharged patients had 102±74 serum C-reactive protein level on day one and 13±11 on day four. The P-value between day four CRP level of expired and discharged patients was 0.006. A weak correlation (r=0.38) was found between raised CRP on day four and death.

## DISCUSSION

COVID-19 pneumonia as a consequence of COVID 19 infection is the leading cause of death in covid. Various drugs are under trial to treat infection and COVID pneumonia but no drug has been effective yet. Remdesivir, Hydroxychloroquine and Lopinavir/Ritonavir combination is not approved by WHO in their clinical trial.^11^

COVID disease is characterized by infectious phase and cytokine storm phase.^12^ Various biological markers are linked with cytokine storm including C-reactive protein. However, COVID-19 have variable course of disease and variable biomarkers in these patients. As a result, different scores based on C-reactive protein level were developed to detect the cytokine storm at an early stage.^13^ one of the predictive scores also included serum albumin level.^14^

We noted in our results that patients came in ICU with normal or near normal serum albumin level, but with increased oxygen requirement developed low serum albumin level side by side within 24 hours. These patients had no reasons like proteinuria or chronic liver disease for low serum albumin level. We also noted that patient developed low serum albumin level along with rising C-reactive protein. This sudden change in Serum albumin level with rising C-reactive protein in four days immediately after admission in hospital ICU is hallmark of COVID-19 pneumonia which is not seen in other Pneumonias. One of the studies also pointed out the low serum albumin level in critically ill patients and related it with multiorgan failure in COVID-19 pneumonia.^15^ However, in our study, we observed sudden change in serum albumin level within four days after admission in intensive care unit. At the same time, these patients have high serum C-reactive protein level. This is the time when patient developed hypoxia, not relieved even by high flow oxygen. So, cytokine storm leads the body metabolism into catabolic state and hypoalbuminemia.

Because sometimes it is difficult to diagnose the COVID-19 disease as PCR in nasopharyngeal swab after one week of symptoms has 40-70 percent sensitivity and normal X-ray chest.^16^ So, it is difficult to diagnose COVID-19 pneumonia where facility of High-resolution CT scan is not available.^17^ This sudden decrease in serum albumin along with rising C-reactive protein level can be used as a clue to diagnose COVID-19 pneumonia.

Those patients who were discharge from ICU had improved serum albumin level and those who died of COVID-19 pneumonia had low serum albumin level. Those patients who respond to Intravenous steroids and cytokine blocking agents also improve serum albumin level without intravenous albumin supplementation.

We noted that many patients had normal CRP level on day four after treatment with IV steroids and cytokine blocking agents as mentioned in Table-III. But some patients had low serum albumin level despite normal CRP level. These patients died of COVID-19 pneumonia despite low CRP level. On the other hand, all those patients whose serum albumin level improved, recovered from COVID-19 pneumonia.

**Table III:** Mean serum CRP of expired and survivors on day one and day four post admission in ICU.

Outcome	Mean Serum CRP day one(mg/dl)	Confidence interval	Mean Serum CRP day four(mg/dl)	Confidence interval
Discharged	104±77.3	78.7-129.3	13±11.1	9.6-16.8
Expired	134±87.0	105.4-162.6	43±50.4	26.4-59.4
p-value	0.1		0.006	

### Limitation of study

Our observations may further be validated by data of a greater number of patients in multicenter studies.

## CONCLUSION

We recommend daily monitoring of serum albumin level as a biological marker for monitoring of cytokine storm and risk of death in COVID-19 pneumonia.

### Authors’ Contribution:

**MAB:** Designed the study, acquired data, drafted the manuscript, approved the final version, agreed to be accountable for all aspects.

**MMR:** Interpreted data, reviewed the manuscript, approved the final version, agreed to be accountable for all aspects.

**MB:** Conceived the study, analyzed data, reviewed the manuscript, approved the final version, agreed to be accountable for all aspects.

**MUB:** Analyzed data, reviewed the manuscript, approved the final version, agreed to be accountable for all aspects.
